# Relationship between Personal Values and Intentions to Purchase Plant-Based Meat Alternatives: Application of the Dual Concern Theory

**DOI:** 10.3390/ijerph19148673

**Published:** 2022-07-16

**Authors:** Ha-Won Jang, Meehee Cho

**Affiliations:** College of Hotel and Tourism Management, Kyung Hee University, Seoul 02447, Korea; janghawon@khu.ac.kr

**Keywords:** egoistic value, biospheric value, social-altruistic value, dual concern theory, plant-based meat alternatives, purchase intentions, generations

## Abstract

This study examines consumers’ intentions to purchase plant-based meat alternatives (PBMA), which have been created to protect animal rights, the global environment, and consumers’ health. Data from 319 Korean consumers were analyzed to establish a causal relationship among personal values (egoistic, biospheric, and social-altruistic), the dual concern theory (anticipated positive effect and empathetic concern), and PBMA purchase intentions. Multigroup analysis was performed for all paths according to generation, divided into Generation MZ and the older generation; “MZ” is a compound term referring to millennials and Generation Z, who have grown up in a digital environment; this collective generation comprises individuals born after 1980. Our analysis revealed that, among personal values, biospheric and social-altruistic values had positive effects on both anticipated positive effect and empathetic concern. In turn, these aspects positively influenced PBMA purchase intentions. Particularly, anticipated positive effect had a strong influence. Finally, a moderating effect was confirmed for two paths, and only Generation MZ demonstrated the enhancing influence of biospheric value on both anticipated positive effect and empathetic concern. This study has several implications and contributes to the sustainable growth and development of PBMA and the overall food service industry.

## 1. Introduction

Plant-based meat alternatives (PBMAs) can protect human health and the environment [1]. Meat has long been the primary source of protein for individuals worldwide [2]; on average, a European consumes 65 kilos of meat per year while an American and a Canadian consume 95 kilos [1]. However, the livestock industry causes problems related to land and water use, and the animals’ bodily processes, such as eructation and flatulence, accelerate the formation of greenhouse gases [2,3]. These environmental problems have contributed to climate change and are a common cause for concern globally [2,3]. The livestock industry is being criticized now more than ever as it is positioned at the intersection of multiple complex issues, such as environmental protection, consumer health, and animal rights protection [4]. Several studies have demonstrated how consuming red meat threatens consumers’ health [5,6]. In addition, as the world’s population continues to increase, a gradual scarcity of food resources, ingredients with high environmental value, and high protein nutrients has set in [7].

With modern consumers seeking alternatives to meat, PBMAs are fast becoming popular [1]. Consumers today are more knowledgeable than in the past; according to Carlsson et al. [1], consumers with higher levels of education are more likely to prefer PBMAs. The primary reason for consuming meat has been protein intake, but PBMAs also have sufficient protein to meet our daily requirement [4]. They contain all essential amino acids and are beneficial for our health [8]. Moreover, the protein digestibility has been confirmed to be more in leguminous products than animal-derived proteins [9].

PBMAs are a future food resource that can replace meat and is produced using plant-based proteins, such as soybeans, wheat, vegetables, and fungus [4]. PBMA foods, beyond alleviating the different types of environmental pollution caused by the livestock industry, are suitable for vegetarians and halal food consumers who shun meat due to religious and ethical beliefs [4]. Globally, attention is being focused on highly promising food resources that can contribute to the sustainable growth and development of the food industry [4,6]. The European market, considered a major PBMA food powerhouse, is expected to grow to 2.4 billion Euros by 2025 [6] while the market in the United States is expected to grow to 21.23 billion USD [5,10].

Lee et al. [11] identified personal values as the most significant influence on consumers’ social responsibility, and consumptive and ecofriendly behaviors with regard to social and environmental phenomena. In general, consumers behave according to their personal values [11,12], which are an expression of their individual preferences and imply their continuing belief [13]. Personal values have been actively utilized for studying consumer behavior in various fields, such as sociology, psychology, and environmental studies [14,15,16]. In particular, this study focuses on Stern and Dietz’s concept of personal value [14], which emphasizes biospheric value.

Butts et al. [17] stated that consumers live in a state of two different emotions continually colliding. These emotions constitute dual concern; one is anticipated positive effect, which prioritizes the benefits one can earn, and the other is empathetic concern, which is the desire to care for others and the society before oneself [17]. This dual concern in consumers’ daily lives shapes their behavior in a specific direction [18]. Sorenson et al. [19] affirm that dual concern is part of every individual’s inner psychology, and, hence, it has been applied in diverse fields. Dual concern also plays a meaningful role in the process of purchasing PBMA food items.

Van Loo et al. [6] found that younger individuals showed more preference for and interest in choosing PBMAs. Carlsson et al. [1] also found that younger individuals with greater adaptability to societal changes prefer PBMAs and, as consumers age, negative perceptions of PBMAs increase. Studies in various fields have identified age and generation as key variables that distinguish consumer behavior [20,21,22]. Therefore, this study aimed to explore the differences in intentions behind PBMA consumption according to generations. Specifically, we divided participants into MZ and older generations; MZ is a compound term used to refer to millennials and Generation Z—that is, individuals born after 1980 who have grown up in a digital environment [22].

This research can contribute to the expansion of the PBMA food industry and significantly impact the sustainable growth and development of the restaurant and hospitality industries. The goals of this study are summarized as follows:

1. To explore the relationship between personal values (egoistic, biospheric, and social-altruistic) and dual concern (anticipated positive effect and empathetic concern);
2. To examine the relationship between dual concern (anticipated positive effect and empathetic concern) and PBMA purchase intentions;
3. To analyze the moderating effects according to generation (MZ and older) to confirm the differences among all the aforementioned paths.

## 2. Theoretical Framework and Hypotheses

### 2.1. PBMA Food Research in the Context of Consumer Behavior

Various analyses of PBMA foods have been conducted in different countries and academic fields; this section shall only summarize content related to PBMA food research in the field of consumer behavior. We aim to conduct research focusing on the field of consumer behavior and have thus organized the contents focusing on previous studies in related fields. Van Loo et al. [6] conducted exploratory research on the differential preferences for meat and PBMAs among American consumers. Their results revealed that 72% of consumers preferred meat with only the remaining 28% choosing PBMAs. Moreover, when the products and the brand were presented together, the proportion of consumers who chose meat increased to 80%. Vegetarians, highly educated consumers, and relatively younger consumers preferred PBMA products; moreover, they stressed upon actively sharing information on PBMAs to increase the consumer base.

Carlsson et al. [1] conducted an exploratory study of Swedish consumers’ intentions to switch from meat to PBMAs. One-third of the participants, who usually liked meat, were willing to choose PBMAs when offered monetary compensation. However, around half of the participants would not choose PBMAs even if provided free of cost. In particular, male consumers aged 30 years or older and without university education had the most negative perception of PBMA. Hence, the researchers argued that an individual’s level of knowledge of environmental and health issues and their choice of PBMAs are highly correlated.

Marcus et al. [23] studied PBMA food-purchasing behavior among German consumers. They analyzed how environmental concerns, health concerns, and animal welfare influenced purchase intentions. It was found that, while health concerns had a direct, significant effect on PBMA purchase intentions, animal welfare had an indirectly significant effect through consumer attitudes, and environmental concerns did not have any significant effect on purchase intentions.

Moreover, while extensive research on PBMAs has been conducted in several countries, most studies have focused on the field of food engineering and nutrition [2,7,24] with mostly qualitative research (such as review and market research) on consumer behavior [25]. Therefore, it is necessary to conduct quantitative research on consumer behavior. Accordingly, this study analyzes consumers’ intentions to purchase PBMAs by applying personal value dimensions and the dual concern theory, thereby expecting to provide a fresh theoretical foundation for related research in the future.

### 2.2. Personal Value

Rokeach [26] defined value as a continuous belief and described it as both a personally and socially acceptable state of being in a particular situation. As a concept, value includes the interaction between individuals and society as well as the social institutions that fulfill biological needs. It can be defined as a universal cognitive representation of humans [27] reflecting a society’s culture and widely shared by its members [13].

Personal values are formed by reflecting upon widely shared cultural elements, norms, beliefs, and values in society; once permeated into humans, values have a subtle yet lasting effect on psychology and attitudes [28]. In general, personal values simultaneously influence various human beliefs and behaviors [29]. Moreover, they are more persistent and stable compared to other psychological variables [12].

Stern and Dietz [14] expanded Schwartz’s concept of personal values [30] by formulating three sub-concepts (egoistic, biospheric, and social-altruistic) and analyzing their influence on human attitudes and behaviors. First, egoistic values are directly related to individual performance and focus almost exclusively on the benefits and well-being to be accrued [14,31]. Second, biospheric values are directly linked to biological and natural environments, and individuals with these values focus on environmental issues and behave in a comparatively more ecofriendly manner [14,31]. Third, those with social-altruistic values emphasize the welfare of others more than their own and perceive others and the natural environment from a moral perspective [14,31,32]. In particular, biospheric value is a new component added by Stern and Dietz [11] and is a crucial concept for identifying responsible behavior by consumers, especially regarding the environment.

Currently, personal values have been primarily applied to the study of ecofriendly behavior [16,31]. They have been an important factor in the analyses of consumer behavior in various fields, such as ecology, anthropology, psychology, and sociology [15,33]. Previous studies have shown how personal values guide an individual’s life [11,12,34]. Moreover, several scholars have reported how personal values drive various human behaviors [12,16,34]. Based on the literature, this study analyzes consumers’ consumptive behavior with a focus on the three personal values mentioned, as presented by Stern and Dietz [14] and Kim and Koo [16].

### 2.3. Dual Concern Theory

Pruitt and Rubin [18] presented the dual concern theory in academia [19]. It analyzes the psychological relationship between personal values and two conflicting emotions—that is, concern for others versus concern for the self. This theory is an expansion and development of Blake and Mouton’s two-dimensional model of conflict [35,36]. Two emotions are simultaneously at work in human psychology; one focuses on personal interests and the other on helping others by excluding personal interests [17]. In every situation, humans experience both these emotions simultaneously [17]. However, they manifest in extremely different patterns depending on the situation an individual is in [19].

We presently explain the two sub-concepts of the dual concern theory in greater detail. First, an individual acts according to what they think would be favorable for them in a particular situation [17]. Meglino and Korsgaard [37] stated that, in general, individuals make decisions by linking them to the expected positive benefits. Diener [38] also argued that, ultimately, individuals act to at least experience positive emotions. Second, individuals’ behaviors might be motivated by altruism, which is called empathetic concern [17] and is based on feelings of empathy and concern for others, acting as a driving force in extending help [39]. In other words, it is an emotion to derive and appropriately provide others with the required welfare [40]. Moreover, those who regard empathetic concern highly do not care much about their personal interests or costs when helping others [41]. They primarily focus on contributing to society [41]. According to Cameron and Payne [42], individuals high in empathetic concern wish to relieve social and environmental issues and maximize their ability to do so.

The major difference between the two sub-concepts of the dual concern theory (anticipated positive effect and empathetic concern) lies in their areas of focus—that is, either personal gains or others’ circumstances [19]. A human’s internal state involves a clash between these emotions at every moment [19]. The dual concern theory has the advantage of being able to clearly describe humans’ inner states in particular contexts [19]. However, despite its importance, it has not been applied widely to research in various fields.

In an analysis conducted by Kim and Koo [16], the dual concern theory was applied to ecofriendly behavioral research. This revealed that humans simultaneously perceive emotions for the benefit of themselves and others through personal values and specific actions. In particular, the biospheric value was found to increase anticipated positive effect, whereas both the biospheric and social-altruistic values were found to increase empathetic concern for others. The analysis presented the two different manifestations of human emotions in all decision-making processes.

This study applies the dual concern theory to analyze how these emotions affect consumers’ intentions regarding purchasing PBMAs. It thus intends to provide a fresh theoretical foundation for related research in the future.

### 2.4. Relationships among the Constructs

#### 2.4.1. Relationship between Personal Values and Anticipated Positive Effect

Egoistic value is focused on personal interests and the inner self rather than others’ feelings or situations [43]. Meglino and Korsgaard [37] stated that an individual’s egoistic value is further maximized when the economic and psychological benefits to be acquired are large. Västfjäll et al. [44] argued that people who value egoistic value consider their own emotions more important than others’. Thus, an individual’s egoistic value is deeply related to the individual’s positive emotion induction [44]. Individual egoistic value appears more vividly as the psychological sense of achievement and expected profits increase [17]. According to Han et al. [45], a person with greater egoistic value is expected to have a higher level of anticipated positive effect for or in a specific object or situation, respectively. Kim and Koo [16] also demonstrated how egoistic value significantly affects anticipated positive effect in the field of tourism.

According to Han [46], the more a person feels responsible for the natural environment or society, the higher their perception of the anticipated positive effect. A deep relationship has been noted between an individual’s biospheric value and anticipated positive effect in terms of the environment [45]. According to Klöckner [47], individuals with biospheric value tend to value not just the harmony of individuals and the environment but also the anticipated positive effect. Kim and Koo [16] also demonstrated how biospheric value significantly affects anticipated positive effect.

Klöckner [47] stated that the more importance individuals place on social altruism, the higher the anticipated positive effect induced by the social environment and harmony with others. Han [46] stated that, as a concept, social-altruistic value focuses on the relationship between the social environment and others, and the greater the value of social altruism, the higher the anticipated positive effect. In addition, Kim and Koo [16] demonstrated how social-altruistic value significantly affects anticipated positive effect.

Thus, based on the results of previous studies on egoistic, biospheric, social-altruistic values, and anticipated positive effect, the following hypotheses were formulated:

**H1.**  *Consumers’ egoistic value positively affects “anticipated positive effect”.*

**H2.**  *Consumers’ biospheric value positively affects “anticipated positive effect”.*

**H3.**  *Consumers’ social-altruistic value positively affects “anticipated positive effect”.*

#### 2.4.2. Relationship between Personal Value and Empathetic Concern

Kim et al. [48] stated that individuals who deeply value nature and their surroundings internalize personal values, which comprise egoistic, biospheric, and social-altruistic values. In other words, empathetic concern for one’s surroundings and the environment is based on personal values, and, hence, personal values and empathic concern can be shown to be intricately linked. Junot et al. [49] argued that individuals with a high personal value are more sympathetic and feel positive emotions for others in their lives. Moreover, empathetic concern is itself stirred by the emotional states of others and can also be explained as the emotional response to others [50].

Logan [51] found that individuals with greater egoistic values are less sensitive to other people’s situations and emotions than individuals with greater biospheric or social-altruistic values. Jones [52] explained that, among the personal values, egoistic value has the weakest effect on empathetic concern. This is because egoistic value emphasizes personal problems instead of that of others. Accordingly, this study reconfirms the relationship between egoistic value and empathetic concern. Additionally, Kim and Koo [13] demonstrated how egoistic value significantly affects empathetic concern.

Butts et al. [17] reported that biospheric value focuses on the situations and welfare of others rather than of oneself; hence, individuals with biospheric value also value empathetic concern. According to Meglino and Korsgaard [37], individuals high in biospheric values are more likely to pursue social and environmental goals connected to empathetic concern without considering their personal costs or benefits. Slovic [53] stated that, the greater the biospheric value, the higher the empathetic concern for others and nature. Han et al. [45] also explained that people with greater biospheric values are more sensitive to empathetic concern. Finally, Kim and Koo [16] showed how biospheric value significantly affects empathetic concern.

Butts et al. [17] explained social-altruistic value as more focused on facilitating a better environment for others than for oneself and, hence, individuals with this value respond more positively to empathetic concern. As with biospheric value, Meglino and Korsgaard [37] stated that individuals high in social-altruistic value do not consider the personal benefits they can accrue but rather pursue environmental and social goals. Slovic [53] stated that the greater the social-altruistic value, the higher the empathetic concern for others and nature. Han et al. [45] also explained that individuals high in social-altruistic value are more aware of and focused on empathetic concern than those with low social-altruistic value. Moreover, Kim and Koo [16] presented how social-altruistic value significantly affects empathetic concern.

Based on previous studies on the relationship between personal value and empathetic concern in connection with the society or the environment, the following hypotheses were formulated:

**H4.**  *Consumers’ egoistic value positively affects “empathetic concern”.*

**H5.**  *Consumers’ biospheric value positively affects “empathetic concern”.*

**H6.**  *Consumers’ social-altruistic value positively affects “empathetic concern”.*

#### 2.4.3. Relationship between Anticipated Positive Effect and PBMA Purchase Intentions

Anticipated positive effect, a component of the dual concern theory, indicates the maximization of an individual’s psychological interests [19]. According to Warr [54], anticipated positive effect is the foundation of individuals’ activities for society and the natural environment. Erlandsson et al. [55] posited that, generally, people are more likely to behave in an environmentally and socially sound manner when there is an anticipated positive effect. Andreychik and Lewis [56] argued that individuals behave as appropriate to a situation based on anticipated positive effect. Finally, Kim and Koo [16] demonstrated that anticipated positive effect significantly affects individuals’ pro-environmental behavior.

Thus, the following hypothesis was formulated:

**H7.**  *Consumer’s anticipated positive effect positively affects “PBMA purchase intention”.*

#### 2.4.4. Relationship between Empathetic Concern and PBMA Purchase Intentions

According to the dual concern theory, empathetic concern comes into play when the intention is purely to help others for social and environmental benefit [56]. Empathetic concern involves caring about social problems, maintaining positive relations with others, and approaching others proactively [56]. Loewenstein and Small [39] explained empathetic concern as the most inspiring factor in an individual’s intention to help others. Woosnam and Norman [57] stated that the more sympathetic and understanding one is of social or environmental issues, the more pro-social one’s behavior is. Woosnam [58] reported that, generally, individuals behave in a more pro-social manner when they empathize with others. In addition, Junot et al. [49] explained that individuals with more empathy for social issues are more active, and Kim et al. [48] asserted that individuals high in social empathy behave more proactively than those with low social empathy. Finally, Kim and Koo [16] presented that individuals’ empathetic concern significantly affects their pro-environmental behavior.

Using this context, the following hypothesis was formulated:

**H8.**  *Consumers’ empathetic concern positively affects “PBMA purchase intention”.*

#### 2.4.5. Moderating Role of Generations (MZ vs. Older)

The generations currently creating and leading new social phenomena and popular cultures worldwide are the millennials and Generation Z [59,60]. They are also referred to as the “digital generation”—that is, a generation that is intricately familiar with the digital world and various electronic devices [22,60]. Millennials include the individuals born between 1980 and 1996 and are also called Generation Y [59]. Generation Z includes those born between 1997 and 2010 [59,60]. Since millennials and Generation Z have grown up in a similar social environment, they are collectively called Generation MZ [60]. According to Jung et al. [22], Generation MZ grew up in the era of globalization, valuing transparent and autonomous communication in society, and with strong egocentrism and self-realization needs. In contrast, Generation X (individuals born between 1965 and 1979) grew up in a society marked by individualism, materialism, and competition, which placed importance on life goals, social growth, and efficiency [22]. Especially with regard to the sociocultural and economic environment, Min and Han [61] argued that the generation to which an individual belongs is the most important psychological variable affecting consumptive behavior and is reflected in their lifestyle and values. Compared to the older generation, Generation MZ shows various differences in their consumption patterns and behaviors [20,21,22].

Kim et al. [20] studied ecofriendly behavior among employees in the hospitality industry by analyzing moderating effects according to Generations X and Y for all paths presented in the study. Their study confirmed a moderating effect in the relationship between autonomous motivation and pro-environmental behavior. A significant effect was found in both groups with Generation X particularly showing a higher influence. Alhouz and Hasouneh [21] also studied the hospitality industry—specifically, the influence of consumers’ social responsibility on civic behavior—and analyzed moderating effects based on generation. They found a significant moderating effect in the relationship between social responsibility and civic behavior and an inverse relationship between respondents’ age and influence of social responsibility on civic behavior (i.e., the older the respondents, the weaker the influence of social responsibility on civic behavior).

**Figure 1:**
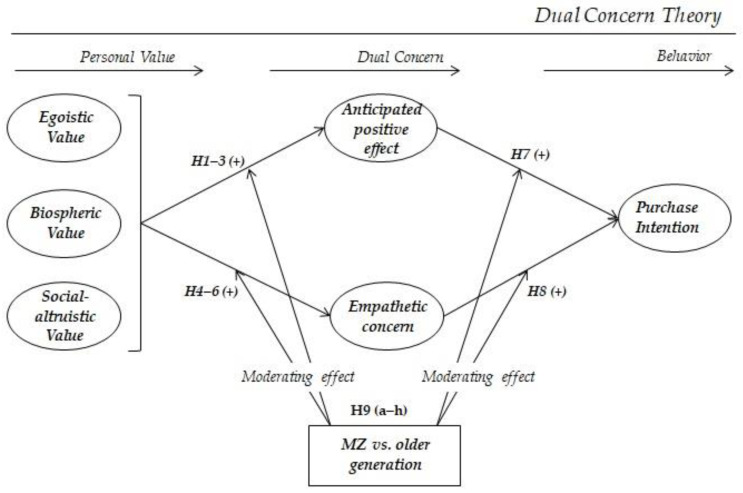
Research framework.

Thus, various studies on the hospitality industry have been conducted to analyze moderating effects based on generation. However, varying results were obtained depending on the research topic and variables. To obtain broader results that can be helpful for future research, this study analyzes the moderating effect based on generation for all paths. Accordingly, the following hypothesis was formulated (see Figure 1):

**H9.**  *There is a moderating effect based on generations (MZ vs. older) for all paths.*

## 3. Materials and Methods

### 3.1. Data Collection and Participants

Data were collected through an online survey of Korean consumers aged 18 years and above. Data collection using online surveys is considered an appropriate way to conduct surveys with reliable panels [62]. The survey was conducted for approximately one week in the first half of January 2022. Once the respondents were told of the study’s purpose via e-mail, the company conducting the online survey clarified that the data would be collected anonymously and used only for academic research. Only the respondents who agreed to this were asked to click on the link listed in the email and participate in the survey. The online survey company paid a certain amount of money to all the participants as a reward.

### 3.2. Survey Instrument and Measures

All the questions on personal values, including egoistic (three questions), biospheric (four questions), and social-altruistic value (three questions), and those on dual concern, including anticipated positive effect (four questions) and empathetic concern (four questions), were adapted from Kim and Koo’s study [16]. However, the last four questions on purchase intention were adapted from the study conducted by Al-Swidi et al. [63]. All the questions were measured using a five-point Likert scale (1 point: highly disagree; 2 points: disagree; 3 points: normal; 4 points: agree; 5 points: highly agree) and translated into Korean before being presented to the respondents. However, at that time, in order to use the measurement questions of prior studies almost as they are, only a minimum number of words were modified. Two professors in the hospitality industry, fluent in both Korean and English, participated in the translation process and confirmed the results.

The survey comprised three stages. The definition of PBMA was presented in the first stage, questions about all the variables of the research model were presented in the second stage, and six demographic questions were presented in the third stage. To analyze the results, reliability, frequency, and descriptive statistical analyses were performed using SPSS version 22, and confirmatory factor, structural equation model (SEM), and multigroup analyses were performed using AMOS version 22 (IBM, Meadville, PA, USA).

## 4. Results

### 4.1. Sample Characteristics

Table 1 presents the respondents’ characteristics. The proportion of men (49.2%) and women (50.8%) was almost equal, with slightly more unmarried respondents (51.7%) than married (47%). Concerning age, the highest percentage of respondents was in their 30s (31.3%), and, when divided into generations, the ratio of MZ generation (58.9%) was higher than that of the older generation (41.1%). In terms of education level, the proportion of four-year university enrollment and graduates was the highest (58.6%). Concerning respondents’ occupations, the proportion of office-goers (45.5%) was the highest. Finally, in terms of monthly income, the ratio of 2000 USD (32.6%) per month was the highest.

### 4.2. Validity and Reliability of Measurements

A confirmatory factor analysis was conducted to confirm convergent validity. The average variance extracted (AVE) values of all the constructs were higher than 0.5, and their composite construct reliability values were higher than 0.7. Therefore, the convergent validity of each construct was satisfied [64]. In addition, the Cronbach’s alpha value, which represents the internal consistency of each construct, was found to be higher than 0.7, confirming internal consistency [64] (See Table 2).

Table 3 presents the results of discrimination validity between the constructs. The values of the diagonal are the square roots of the AVE values while those at the bottom of the diagonal are correlation coefficients. Comparing the square roots of the AVEs and the correlation coefficients showed that all correlation coefficients were lower than those of the square roots of the AVEs. Therefore, discriminant validity was also confirmed [64] (see Table 3).

### 4.3. Results of Hypotheses 1 to 8

An SEM analysis was conducted to confirm the relationships between personal values (egoistic, biospheric, and social-altruistic), dual concern (anticipated positive effect and empathetic concern), and PBMA purchase intentions. Personal values significantly influenced consumers’ anticipated positive effect. While biospheric (β = 0.376; *p* < 0.000) and social-altruistic values (β = 0.367; *p* < 0.000) positively influenced anticipated positive effect, egoistic value influenced it negatively (β = −0.148; *p* < 0.05). Therefore, H2 and H3 were supported, but H1 was not.

Furthermore, biospheric (β = 0.566; *p* < 0.000) and social-altruistic values (β = 0.253; *p* < 0.01) significantly and positively influenced empathetic concern, but egoistic value did not influence it significantly (β = 0.060; *p* > 0.05). Therefore, H5 and H6 were supported, but H4 was not. Moreover, both the anticipated positive effect (β = 0.640; *p* < 0.000) and empathetic concern (β = 0.214; *p* < 0.000) of consumers positively influenced PBMA purchase intentions. Therefore, H6 and H7 were supported.

The values for the model fit were χ^2^/df = 2.282 ***, root mean square residual = 0.040, goodness of fit index = 0.888, Tucker–Lewis index = 0.941, comparative fit index = 0.950, incremental fit index = 0.950, and root mean-square error of approximation = 0.063. No issues were observed in terms of model fit [64] (see Table 4).

### 4.4. Results of Hypothesis 9

A multigroup analysis was performed to confirm the difference between the two groups (Generation MZ vs. older generation). There were 188 respondents in the Generation MZ group and 131 in the older generation group. The results showed a significant difference between the two groups in a total of three paths. However, in the H9a path, the analysis of the moderating effect was meaningless because H1(=H9a) was already rejected in the SEM analysis. Therefore, excluding H9a, a moderating effect was interpreted only for the other two paths.

First, a moderating effect was confirmed in the relationship between biospheric value and consumers’ anticipated positive effect. Specifically, only Generation MZ was found to have positively increased anticipated positive effect through biospheric value (β = 0.472; *p* < 0.000); no significant results were found for the older group (β = −0.023; *p* > 0.05). Therefore, H9b was supported. A moderating effect was also confirmed in the relationship between biospheric value and empathetic concern. For this path as well, only Generation MZ demonstrated positively enhanced empathetic concern through biospheric value (β = 0.589; *p* < 0.000) with no significant results for the older group (β = 0.213; *p* > 0.05). Therefore, H9e was supported (See Table 5).

## 5. Discussion

### 5.1. Theoretical Implications

This study has several academic implications. First, it derived meaningful results by applying dimensions of personal values and the dual concern theory, for the first time to the best of our knowledge, to the study of PBMA consumption behavior. Research on PBMA consumption behavior has not yet been actively conducted, and this study is the first to apply the dual concern theory to research on PBMA consumption behavior.

Second, this study revealed the relationship between personal values, comprising three sub-concepts (egoistic, biospheric, and social-altruistic), and the dual concern theory, comprising two sub-concepts (anticipated positive effect and empathetic concern). Only biospheric and social-altruistic values were found to have a positive effect on consumers’ anticipated positive effect. Between the two values, the influence of biospheric value was slightly stronger. However, egoistic value was found to have a negative effect on consumers’ anticipated positive effect, and the related hypothesis was rejected. Han et al. [45] explained that individuals with greater egoistic value would also demonstrate high anticipated positive effect, but the results of this study revealed the opposite. In other words, it was confirmed that consumers high in egoistic value did not have a positive outlook toward anticipated positive effect as it focuses only on personal interests and satisfaction in terms of PBMA consumption. Kim and Koo [16] also found a negative impact of individuals’ egoistic value on anticipated positive effect. Even with the same subject, it is common for the results derived to vary depending on the field or situation of the research [65].

Third, this study is significant as it supports the relationship between personal values and empathetic concern. We found that consumers’ biospheric and social-altruistic values had a positive effect on empathetic concern. According to Slovic [53], individuals with greater biospheric values show more empathetic concern for the natural environment or society. Butts et al. [17] also explained that individuals who emphasize biospheric value in their lives show more empathetic concern. This study confirmed their findings that consumers with greater biospheric value show greater empathetic concern. However, egoistic value did not significantly affect empathetic concern. According to Logan [51] and Jones [52], among the sub-concepts of personal values, egoistic value has the weakest influence on empathetic concern. This is because individuals with greater egoistic value focus more on themselves than on others and society. Similar results were obtained in this study. Thus, it is difficult to affirm any close relationship between egoistic value and empathetic concern.

Fourth, this study confirms the relationship between the dual concern theory and PBMA purchase intentions, revealing that both anticipated positive effect and empathetic concern had a positive effect on PBMA purchase intentions. Particularly, anticipated positive effect had a much stronger influence. A study by Kim and Koo [16] on the ecofriendly behavior of tourists found that anticipated positive effect had a much higher influence on ecofriendly behavior than empathetic concern. Thus, this study confirms the strong causal relationship between anticipated positive effect and PBMA purchase intentions.

Finally, this study analyzed moderating effects based on generations (MZ vs. older) for all the paths presented in the research model and obtained insightful results. As previously mentioned, the SEM analysis showed that, among the sub-concepts of personal value, biospheric value had the strongest influence on anticipated positive effect and empathetic concern. The analysis further identified moderating effects in two paths related to biospheric value. A moderating effect was observed in the relationships of biospheric value with anticipated positive effect and empathetic concern. Both paths showed similar patterns. However, the positive effect of biospheric value on anticipated positive effect and empathetic concern was demonstrated only in Generation MZ; it had no statistical significance in the older generation. In other words, Generation MZ, which is relatively young and will constitute key members of society in the near future, showed a significant positive effect on dual concern through biospheric value. This finding is extremely relevant because it is intricately linked to the possibility of more developed social environments in the future.

### 5.2. Managerial Implications

This study also has several implications for managerial contexts. First, research on PBMA consumption behavior has been actively conducted only in the fields of nutrition and food engineering, and much of it has not analyzed consumer behavior. PBMA consumption behavior requires further proactive research in the future, and this study is rendered all the more meaningful by its several implications for research in various related fields.

Second, this study showed that consumers with greater biospheric and social-altruistic values also demonstrated greater anticipated positive effect. As concepts, both values emphasize the individual’s environment or the welfare of others rather than the individual. Thus, it can be interpreted that consumers with greater consideration for the natural environment, society, and others are more strongly influenced by anticipated positive effect in choosing PBMA. To further increase the purchasing power of PBMA high in biospheric and social-altruistic values, it is necessary to recognize the rationality and positivity of choosing PBMA by presenting to consumers various health- and environment-related information. In their research, van der Weele et al. [2] explained how PBMA production and consumption improves the global environment and increases sustainability in various aspects. However, consumers with greater egoistic value showed a negative influence on anticipated positive effect. To lower egoistic value among consumers, marketing teams must carefully understand what such consumers seek from PBMA foods and make efforts to obtain their suggestions for improvements and solutions.

Third, this study found that consumers with greater biospheric and social-altruistic values also show greater empathetic concern. The influence of biospheric value was particularly high, with similar results obtained by Kim and Koo [16]. From a comprehensive perspective, this study found that, among the three sub-concepts of personal value, biospheric value exercised the strongest influence. This can be interpreted as modern consumers considering biospheric value as an especially crucial personal value. Consumers with greater biospheric value are more interested in others and the society; thus, marketing teams must actively appeal for positive changes in the society and environment that can be caused by consumers choosing PBMA.

Fourth, we found that dual concern had a positive effect on PBMA purchase intentions. In particular, anticipated positive effect had a stronger influence on PBMA purchase intentions than empathetic concern. This can be interpreted as consumers demonstrating higher purchasing power when they expect to benefit from purchasing PBMA. Zhao et al. [66] analyzed modern consumers’ demands for PBMA and found that consumers are benefitted in terms of the environment, health, and economy. Since PBMA is produced from plant-based materials, it can be purchased at a more reasonable price, reduce environmental pollution, and improve consumers’ diet and health [66]. PBMA food marketing teams must extol the advantages of PBMA in various ways and promote it actively by providing information about its positive effects in engaging ways, such as by involving popular figures. Moreira et al. [67] indicated that social media use is effective in maintaining a positive relationship between PBMA manufacturers and consumers. Willett et al. [3] stressed that the government should strongly regulate advertising and marketing for unhealthy and unsustainable foods and provide consumer education on healthy diets that promote the consumers’ health and sustainability of the environment.

Lastly, analysis of the moderating effects based on generations revealed that the greater the biospheric value, the stronger the influence on both anticipated positive effect and empathetic concern. According to Jung et al. [22], Generation MZ leads their lives more actively than the older generation and expresses themselves more transparently and rationally. Thus, to develop PBMA sales strategies, it is essential to conduct an intensive analysis of Generation MZ. This is because this generation is the core of modern society and bases their consumptive behavior on clearer subjective grounds. Therefore, to specifically target Generation MZ, marketing teams should adopt a continuous and long-term perspective to make the purchase of PBMA products a choice for positively changing the ecosystem and the surrounding environment. In Korea, PBMA-related products are gradually diversifying and specialty restaurants are increasing. In this context, consumers’ high preference for PBMA products will eventually contribute to the development of the food service industry. It will also expand the options for halal and kosher consumers, who are restricted from eating meat due to religious reasons. Furthermore, smart food consumption by consumers will eventually contribute to protecting the environment around the world.

## 6. Conclusions

In terms of individual health and the environment, this study supported the relationship between consumers’ personal value and dual concern, which has recently attracted worldwide attention. We found that, among the personal values, the influence of biospheric value was prominent. Its influence was also found on dual concern. Simply put, the causal relationship between biospheric value and dual concern was the strongest. These results indicate that modern consumers are well aware of the importance of nature and protecting the environment. Therefore, various research, products, and marketing strategies that can further satisfy consumers’ biospheric values by comprehensively reflecting their psychology and current trends should be developed. Moreover, we found that Generation MZ considers biospheric value especially important. It is necessary to develop and introduce PBMA products by considering this psychology of Generation MZ, who shall be key members of future society. The fact that they value the environment and nature more than the older generation is an extremely hopeful finding in terms of a brighter future for society at large. We hope that a better environment will be created in the future through the growth and development of the PBMA market. This will eventually contribute positively to the development and growth of the sustainable food service industry.

## 7. Limitations and Future Research

This study has certain limitations. First, as it targeted only Korean consumers, the generalizability of the results remains limited. Second, the study was conducted by adopting three sub-concepts of personal values from previous studies; however, personal values can be subdivided in other ways as well. In this respect, it is necessary to perform future research substituting this concept of personal values with other dimensions. Third, the analysis of the moderating effects based on generations performed in this study divided consumers into Generation MZ (born after 1980) and the older generation (born before 1979). However, it should be recognized that this classification of generation is closely related to the timeline of the spread of the Internet, and this reference point for the classification is not absolute. This is because the period in which Internet use spreads varies across countries.

## Figures and Tables

**Table 1:** Sample socio demographic characteristics (*n* = 319).

Characteristics	*n* (%)	Characteristics	*n* (%)
Sex		Occupation	
Men	157 (49.2%)	Student	36 (11.3%)
Women	162 (50.8%)	Office job	145 (45.5%)
Marital status		Self-employed	21 (6.6%)
Unmarried	165 (51.7%)	Professional job	61 (19.1%)
Married	150 (47%)	Homemaker	31 (9.7%)
Other	4 (1.3%)	Other	25 (7.8%)
Age (Years)		Monthly Income	
20–29	82 (25.7%)	≤$1000	30 (9.4%)
30–39	100 (31.3%)	$1001–$2000	38 (11.9%)
40–49	94 (29.5%)	$2001–$3000	104 (32.6%)
50–59	33 (10.3%)	$3001–$4000	53 (16.6%)
Over 60	10 (3.2%)	$4001–$5000	44 (13.8%)
Generation		≥$5001	50 (15.7%)
MZ	188 (58.9%)		
Older	131 (41.1%)		
Educational level			
High school	41 (12.9%)		
Two-year college	53 (16.6%)		
University	187 (58.6%)		
Graduate school	38 (11.9%)	Total	319 (100%)

Note: Monthly income was calculated as KRW 1232 using the April exchange rate standard.

**Table 2:** Confirmatory factor analysis test results.

Construct	Standardized Loadings	*t*-Value	CCR	AVE	Cronbach’s Alpha
Egoistic Value			0.908	0.766	0.905
I value my authority in life	0.934	Fixed			
I value the influence I have over others	0.861	21.584 ***			
I think my social power is important	0.828	20.176 ***			
Biospheric Value			0.887	0.663	0.885
I value the nature around me	0.842	Fixed			
I value the environment	0.767	15.857 ***			
I think preventing environmental pollution is important	0.834	17.714 ***			
I think environmental protection is important	0.813	17.067 ***			
Social-Altruistic Value			0.774	0.534	0.769
I think world peace is important	0.775	Fixed			
I value justice in society	0.726	11.980 ***			
I want to be a helpful person to society	0.688	11.381 ***			
Anticipated Positive Effect			0.924	0.753	0.924
If I choose PBMAs, I will be proud	0.866	Fixed			
If I choose PBMAs, I will be happy	0.821	18.770 ***			
If I choose PBMAs, I will be satisfied	0.904	22.380 ***			
If I choose PBMAs, I will feel good	0.879	21.263 ***			
Empathetic Concern			0.845	0.577	0.843
When I see nature being taken advantage of, I feel kind of protective toward it	0.822	Fixed			
The nature’s pain usually disturbs me a great deal	0.731	13.960 ***			
When I see nature being treated poorly, I sometimes feel a lot of pity for it	0.788	15.364 ***			
I am often quite touched by things that I see happening around me	0.692	13.031 ***			
Purchase Intention			0.915	0.729	0.913
I am willing to buy PBMAs	0.843	Fixed			
I am willing to buy PBMAs in the future	0.890	20.631 ***			
I am willing to buy PBMAs on a regular basis	0.887	20.374 ***			
I would also recommend others buy PBMAs	0.792	16.956 ***			

Notes: χ^2^/df = 2.121, *p* < 0.001; root mean square residual = 0.030; goodness of fit index (GFI) = 0.897; adjusted GFI = 0.864; Tucker–Lewis index = 0.948; comparative fit index = 0.957; incremental fit index = 0.957; root mean square error of approximation = 0.059; CCR: composite construct reliability; AVE: average variance extracted; *** *p* < 0.001.

**Table 3:** Discriminant validity and correlations.

Construct	1	2	3	4	5	6	Mean	SD
1. Egoistic Value	0.875 ^a^						3.790	0.594
2. Biospheric Value	0.443 ^b^	0.814 ^a^					3.869	0.638
3. Social-Altruistic Value	0.501 ^b^	0.704 ^b^	0.730 ^a^				3.799	0.627
4. Anticipated Positive Effect	0.212 ^b^	0.559 ^b^	0.551 ^b^	0.867 ^a^			3.481	0.846
5. Empathetic Concern	0.452 ^b^	0.775 ^b^	0.673 ^b^	0.583 ^b^	0.759 ^a^		4.000	0.630
6. Purchase Intention	0.208 ^b^	0.428 ^b^	0.323 ^b^	0.756 ^b^	0.602 ^b^	0.853 ^a^	3.431	0.876

Note: ^“a”^ diagonal elements are the root of the average variance extracted; ^“b”^ bottom diagonal elements are the correlations.

**Table 4:** Results of Hypotheses 1 to 8.

	Relationships	β	B	S.E.	*t*-Value	*p*-Value	Results
H1	Egoistic Value → APE	−0.148	−0.190	0.081	−2.343 *	0.019	Not Supported
H2	Biospheric Value → APE	0.376	0.491	0.113	4.336 ***	0.000	Supported
H3	Social-Altruistic Value → APE	0.367	0.567	0.151	3.743 ***	0.000	Supported
H4	Egoistic Value → EC	0.060	0.060	0.055	1.084	0.279	Not Supported
H5	Biospheric Value → EC	0.566	0.568	0.080	7.063 ***	0.000	Supported
H6	Social-Altruistic Value → EC	0.253	0.301	0.101	2.973 **	0.003	Supported
H7	APE → PI	0.640	0.653	0.059	11.036 ***	0.000	Supported
H8	EC → PI	0.214	0.284	0.070	4.062 ***	0.000	Supported

Notes: APE: Anticipated Positive Effect; EC: Empathetic Concern; PI: Purchase Intention; χ^2^/df = 2.282, *p* < 0.001; root mean square residual = 0.040; goodness of fit index = 0.888; Tucker–Lewis index = 0.941; comparative fit index = 0.950; incremental fit index = 0.950; root mean-square error of approximation = 0.063; * *p* < 0.05; ** *p* < 0.01; *** *p* < 0.001.

**Table 5:** Results of Hypothesis 9.

	Structural Relationship	MZ (*n* = 188)		Older (*n* = 131)		Free	Constrained	Δχ^2^	Results
		β	*t*-Value	β	*t*-Value	χ^2^ (df = 380)	χ^2^ (df = 381)		
H9a	EV → APE	−0.257	−3.188 **	0.072	0.702	733.086	739.347	6.261	Supported
H9b	BV → APE	0.472	4.764 ***	−0.023	−0.117	733.086	738.062	4.976	Supported
H9c	SV → APE	0.344	3.027 **	0.625	2.867 **	733.086	733.412	0.326	Not Supported
H9d	EV → EC	0.086	1.450	−0.008	−0.085	733.086	733.818	0.732	Not Supported
H9e	BV → EC	0.589	7.506 ***	0.213	1.141	733.086	737.028	3.942	Supported
H9f	SV → EC	0.213	2.573 *	0.505	2.502 *	733.086	733.774	0.688	Not Supported
H9g	APE → PI	0.671	9.210 ***	0.564	6.513 ***	733.086	733.387	0.301	Not Supported
H9h	EC → PI	0.240	3.726 ***	0.191	2.320 *	733.086	733.120	0.034	Not Supported

Notes: EV: Egoistic Value; BV: Biospheric Value; SV: Social-Altruistic Value; APE: Anticipated Positive Effect; EC: Empathetic Concern; PI: Purchase Intention; * *p* < 0.05; ** *p* < 0.01; *** *p* < 0.001.

## Data Availability

Not applicable.
